# Study protocol of European Fans in Training (EuroFIT): a four-country randomised controlled trial of a lifestyle program for men delivered in elite football clubs

**DOI:** 10.1186/s12889-016-3255-y

**Published:** 2016-07-19

**Authors:** Femke van Nassau, Hidde P. van der Ploeg, Frank Abrahamsen, Eivind Andersen, Annie S. Anderson, Judith E. Bosmans, Christopher Bunn, Matthew Chalmers, Ciaran Clissmann, Jason M. R. Gill, Cindy M. Gray, Kate Hunt, Judith G.M. Jelsma, Jennifer G. La Guardia, Pierre N. Lemyre, David W. Loudon, Lisa Macaulay, Douglas J. Maxwell, Alex McConnachie, Anne Martin, Nikos Mourselas, Nanette Mutrie, Ria Nijhuis-van der Sanden, Kylie O’Brien, Hugo V. Pereira, Matthew Philpott, Glyn C. Roberts, John Rooksby, Mattias Rost, Øystein Røynesdal, Naveed Sattar, Marlene N. Silva, Marit Sorensen, Pedro J. Teixeira, Shaun Treweek, Theo van Achterberg, Irene van de Glind, Willem van Mechelen, Sally Wyke

**Affiliations:** Department of Public and Occupational Health, and EMGO Institute for Health and Care Research, VU University Medical Center, Van der Boechorststraat 7, Amsterdam, 1081 BT The Netherlands; Norwegian School of Sport Sciences, Department of Coaching and Psychology, Oslo, Norway; Centre for Public Health Nutrition Research, Level 7, Ninewells Medical School, University of Dundee, Dundee, UK; Department of Health Sciences and the EMGO Institute for Health and Care Research, Faculty of Earth and Life Sciences, Vrije Universiteit, Amsterdam, The Netherlands; Institute of Health and Wellbeing, College of Social Sciences, University of Glasgow, Glasgow, G12 8RS UK; School of Computing Science, University of Glasgow, Glasgow, G12 8RZ UK; Pintail Ltd, 77 Springhill Ave, Blackrock, Co. Dublin, Ireland; Institute of Cardiovascular and Medical Sciences, University of Glasgow, Glasgow, G12 8TA UK; MRC/CSO Social and Public Health Sciences Unit, Institute of Health and Wellbeing, University of Glasgow, Glasgow, UK; University of California Santa Barbara, Santa Barbara, CA 93106 USA; PAL Technologies Ltd, PAL Technologies Ltd 50 Richmond Street, Glasgow, G1 1XP Scotland UK; Robertson Centre for Biostatistics, Institute of Health and Wellbeing, University of Glasgow, Scotland, UK; Physical Activity for Health Research Centre, University of Edinburgh, Institute for Sport, Physical Education and Health Sciences, Edinburgh, EH8 8AQ UK; Radboud university medical center, Radboud Institute for Health Sciences, Scientific Center for Quality of Healthcare (IQ healthcare), Nijmegen, The Netherlands; Interdisciplinary Center for the Study of Human Performance (CIPER), Faculty of Human Kinetics, University of Lisbon, Estrada da Costa, 1495-688 Cruz Quebrada, Portugal; European Healthy Stadia Network, 151 Dale Street, Liverpool, L2 2JH UK; Health Services Research Unit, University of Aberdeen, Aberdeen, AB25 2ZD UK; Department of Public Health and Primary Care, Academic Centre for Nursing and Midwifery, KU Leuven, Leuven, Belgium

**Keywords:** Intervention, Randomised controlled trial, Sedentary behaviour, Physical activity, Diet, Long-term behaviour change, Men’s health, Football club, Cardio-metabolic health, Obesity

## Abstract

**Background:**

Lifestyle interventions targeting physical activity, sedentary time and dietary behaviours have the potential to initiate and support behavioural change and result in public health gain. Although men have often been reluctant to engage in such lifestyle programs, many are at high risk of several chronic conditions. We have developed an evidence and theory-based, gender sensitised, health and lifestyle program (European Fans in Training (EuroFIT)), which is designed to attract men through the loyalty they feel to the football club they support. This paper describes the study protocol to evaluate the effectiveness and cost-effectiveness of the EuroFIT program in supporting men to improve their level of physical activity and reduce sedentary behaviour over 12 months.

**Methods:**

The EuroFIT study is a pragmatic, two-arm, randomised controlled trial conducted in 15 football clubs in the Netherlands, Norway, Portugal and the UK (England). One-thousand men, aged 30 to 65 years, with a self-reported Body Mass Index (BMI) ≥27 kg/m^2^ will be recruited and individually randomised. The primary outcomes are objectively-assessed changes in total physical activity (steps per day) and total sedentary time (minutes per day) at 12 months after baseline assessment. Secondary outcomes are weight, BMI, waist circumference, resting systolic and diastolic blood pressure, cardio-metabolic blood biomarkers, food intake, self-reported physical activity and sedentary time, wellbeing, self-esteem, vitality and quality of life. Cost-effectiveness will be assessed and a process evaluation conducted.

The EuroFIT program will be delivered over 12 weekly, 90-minute sessions that combine classroom discussion with graded physical activity in the setting of the football club. Classroom sessions provide participants with a toolbox of behaviour change techniques to initiate and sustain long-term lifestyle changes. The coaches will receive two days of training to enable them to create a positive social environment that supports men in engaging in sustained behaviour change.

**Discussion:**

The EuroFIT trial will provide evidence on the effectiveness and cost-effectiveness of the EuroFIT program delivered by football clubs to their male fans, and will offer insight into factors associated with success in making sustained changes to physical activity, sedentary behaviour, and secondary outcomes, such as diet.

**Trial registration:**

ISRCTN: 81935608. Registered 16 June 2015.

## Background

Low levels of moderate to vigorous physical activity, high level of sedentary behaviour and poor diet are major threats to public health. Low levels of moderate to vigorous physical activity are associated with increased risk of cardiovascular disease, some cancers (breast and colon in particular) and type 2 diabetes [1, 2]. Sedentary behaviour (any waking activity characterised by an energy expenditure ≤1.5 metabolic equivalents and a sitting or reclining posture [3]) is also associated with adverse health outcomes and increased mortality, independent of time spent being physically active [4–7]. However, the health risks of high levels of sedentary time are often not recognised and are poorly understood by the general public. Our recent meta-analysis demonstrated that interventions focussing primarily on physical activity have little effect on sedentary time, whereas those focussing holistically on a combination of physical activity, dietary and sedentary behaviours are more successful in reducing sedentary time [8]. In addition, behavioural interventions that target physical activity as well as diet are also more likely to result in long-term changes in these health behaviours and maintenance of weight loss [9].

Because poor physical activity, dietary and sedentary behaviours all contribute to increased risks for many of the same health outcomes, combined lifestyle intervention programs have the potential to have a substantial public health impact. However, men tend to be underrepresented in lifestyle change programs, such as weight management programs [10], and are often considered a high-risk, but hard-to-reach or underserved group. Men also have higher risk of diabetes and mortality risks than women at the same levels of obesity [11]. In response to this, the gender-sensitised Football Fans in Training (FFIT) program was specifically designed to attract overweight and obese men (aged 35–65) to a program delivered through the top football clubs in Scotland to support men in losing weight, becoming more active and improving their diet [12]. FFIT was successful in recruiting men at high risk of ill health from across the socio-economic spectrum; many reported that the football club setting was a powerful draw in attracting them to the program [13]. A randomised controlled trial (RCT) of FFIT showed that mean weight loss at 12 months was 4.9 kg (95 % CI 4.0, 5.9) or 4.4 % (3.6, 5.1) greater in the intervention group than the comparison group [14]. There were also significant between-group differences in self-reported physical activity and dietary changes at 12 months, also in favour of the intervention group. The process evaluation showed that the group setting (being with other ‘men-like-me’) facilitated these behavioural changes [15].

European Fans in Training (EuroFIT) builds on the success of FFIT, and uses the allegiance that many men have for top professional football clubs in the Netherlands, Portugal, Norway and the UK (England) to attract at-risk men to engage in lifestyle changes. EuroFIT extends the focus of FFIT from weight loss, physical activity and diet to include a reduction in sedentary time. It makes a more explicit and extensive use of theory to support sustained lifestyle modifications. It incorporates a novel device (the SitFIT™) that allows real-time self-monitoring not only of physical activity (through step counts), but also of sedentary behaviour (sitting time) and non-sedentary behaviour (upright time). Finally, participants are also encouraged to use an app-based game (MatchFIT), designed as part of the EuroFIT study, to encourage social support around physical activity between sessions and after the end of the program.

This paper describes the protocol for a randomised controlled trial, which aims to evaluate the effectiveness and cost-effectiveness of the EuroFIT program in supporting men to improve their lifestyles versus a waiting list comparison group that is offered the program after the 12-month follow-up. The primary aim of the trial is to determine whether EuroFIT can help men aged 30–65 years with a self-reported Body Mass Index (BMI) ≥27 kg/m^2^ to increase their physical activity and decrease their sedentary time over 12 months. Secondary outcomes are weight, BMI, waist circumference, resting systolic and diastolic blood pressure, cardio-metabolic blood biomarkers (e.g. glucose, insulin, HbA1c, lipids and liver function), food intake, self-reported physical activity and sedentary time, wellbeing, self-esteem, vitality and quality of life. Cost-effectiveness will be assessed and a process evaluation conducted.

## Methods

### Study design

**Fig. 1:**
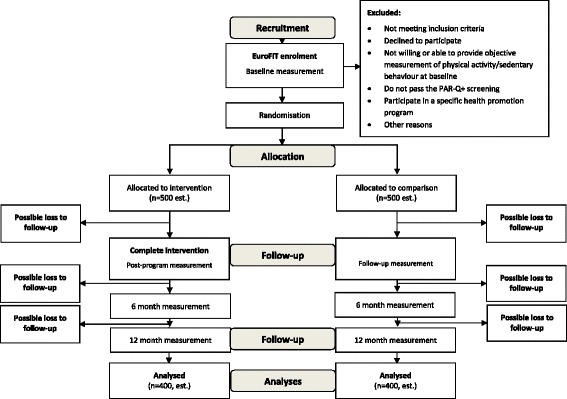
Projected trial profile

This study is a pragmatic two-arm randomised controlled trial to assess the effect of the EuroFIT program in four European countries. The trial will be conducted at 15 football clubs in the Netherlands (four clubs), Norway (three clubs), Portugal (three clubs) and the UK (England; five clubs). In total, 1000 participants will be recruited. Figure 1 summarises the study design using the CONSORT template.

The study was approved in each country by local ethics committees before the start of the EuroFIT study (Ethics committee of the VU University Medical Center (2015.184); Regional committees for medical and health research ethics, Norway (2015/1862); Ethics Council of the Faculty of Human Kinetics, University of Lisbon (CEFMH 36/2015); Ethics Committee at the University of Glasgow College of Medicine, Veterinary and Life Sciences (UK) (200140174)).

### Participants

Evidence from the process evaluation conducted as part of the FFIT RCT suggested that one of the factors that attracted men to the program initially, and engaged them from the outset, was the recognition it attracted other men ‘like me’, both in terms of appearance (e.g., size, shape, level of fitness) as well as their interest in and allegiance to their football club [13, 15, 16]. In order to maximise the chances that men signing up for EuroFIT will also have a sense of being with others who were sufficiently ‘like me’, whilst maximising reach, male football fans aged 30 to 65, with a self-reported BMI ≥27 kg/m^2^ at initial screening will be eligible for inclusion. Since a healthy lifestyle is beneficial for most people, including those with chronic health conditions, recruitment is aimed to be inclusive.

Inclusion criteria:

- Men;
- aged 30–65 years;
- self-reported BMI ≥27 kg/m^2^ at initial screening;
- consent to randomisation.

Exclusion criteria:

- do not provide at least 4 days of usable data from objective measurement of physical activity/sedentary time over the course of one week (as measured by ActivPAL™ from PAL technologies) at baseline;
- have a contraindication to moderate intensity physical activity as assessed by the adapted Physical Activity Readiness Questionnaire-Plus (PAR-Q+) [17];
- are already participating in a specific health promotion program at the club at the time of screening.

### European Fans in Training (EuroFIT) program

The EuroFIT program is designed to support men to: become more physically active and less sedentary; improve their diet; and maintain these changes over the long term. The EuroFIT program will be delivered over 12, weekly, 90-minute sessions that combine classroom discussion with graded group-based physical activity led by community coaches, with one reunion meeting held 6–9 months after the program ends. The EuroFIT program has built on the weight management, physical activity and healthy eating components used in the FFIT program [12], but extends FFIT in the following ways:

- EuroFIT incorporates a specific focus on reducing sedentary time through the integration of a novel pocket-worn technology (the SitFIT developed by PAL technologies) for self-monitoring of sedentary and non-sedentary time and a greater focus on sedentary time in the classroom discussion;
- EuroFIT focuses on physical activity, sedentary behaviour and healthy eating, rather than weight loss (although this is encouraged where appropriate);
- EuroFIT aims to promote sustained lifestyle change by:
  - o drawing more explicitly on motivational theories (Self-Determination Theory [18] and Achievement Goal Theory [19]) to encourage men to develop internalised and self-relevant motivation for becoming more active, sitting less and eating a healthier diet;
  - o further supporting men to develop self-regulation strategies that increase the value and importance of health behaviours for their own lives [20];
  - o providing even greater emphasis on relapse prevention techniques [21];
  - o embedding between-session and post-program peer support for changing behaviour through social media and game-based social interaction (the MatchFIT app);
- EuroFIT is culturally-sensitised for the different countries to reflect local physical activity and dietary norms.

Like FFIT, the program is gender-sensitised in relation to context, content and style of delivery. In relation to context, delivery through top professional football clubs aims to attract men either by tapping into the powerful loyalty and affiliation that many feel (as self-identified football fans) towards the club they support, or by providing the opportunity to take part in a program in a context that men are likely to see as unthreatening to male identities. In addition, the EuroFIT coaches will be trained in creating a positive social environment that supports men in making changes suited to their own routines and preferences.

In relation to content, EuroFIT explicitly targets theory-derived mechanisms of action (e.g. autonomous motivation, task-oriented goals), makes use of the most evidence-based self-regulation techniques (e.g. self-monitoring, goal setting, implementation intentions) [22] and is also informed by sociological theory [15] and how gendered identities relate to health behaviours [23]. Using the supporting manual developed by the EuroFIT consortium, coaches will provide participants with a toolbox of behaviour change techniques, which are reinforced and practised through interaction and discussion between participants during face-to-face group sessions. The materials are designed to help participants to embed the new behaviours into their everyday life so that they are able to maintain these changes in the long term. Participants choose from the skills and strategies in the EuroFIT toolbox to change their physical activity, sedentary behaviour and diet. Simple, practical, relevant messages allow participants to understand what they can do to personally improve their physical activity, sedentary behaviour and diet. The men are supported to choose to engage in the behaviours that personally fit in their life and to develop a clear rationale for why they value these new behaviours. Moreover, interactions with other participants provide support to collaboratively tackle challenges and encourage changes being made. Together these components foster the formation of new, self-endorsed, healthy lifestyle routines that sustain behaviour change.

Self-monitoring of physical activity with a pedometer is an effective strategy to improve physical activity behaviour [24, 25], and proved to be very popular amongst men as one element of the FFIT program [23]. In light of this, EuroFIT has developed the SitFIT, a pocket-worn activity and sedentary/non-sedentary behaviour monitor. The SitFIT provides real time feedback on both step counts *and* upright (non-sedentary) time and so allows participants to actively self-monitor their daily physical activity (steps), sitting time and upright time (time spent standing and walking). Participants use their SitFIT to track their progress against an individualised, incremental program to increase both their daily step count and time spent upright. The SitFIT can also display steps and upright time data over the past seven days, and each participant can obtain a more detailed historical record of his SitFIT data via computer upload (PC and MAC) to the MatchFIT app. MatchFIT has been developed as part of the EuroFIT study to enable between-session social support via a chat function, and provides a competitive element where each club-based EuroFIT group can compete collectively in a step challenge against a computer-generated football team, using an algorithm which takes account of the group’s previous week’s step performance. It should be noted that the competitive element is not a person-to-person competition. Rather, the competition is group-based to enhance the social support aspect of the program.

In relation to style of delivery, EuroFIT-licensed coaches (who receive two days of standardized training to deliver EuroFIT) help the men feel comfortable and receptive to change from the outset by reinforcing the experience that they are with other ‘men-like-me’ and that their efforts each week, within and between program sessions, are valued by the coach and the club. In particular, the coaches are instructed in how to create a motivational, and autonomy- and mastery-supportive climate, and on the importance of understanding and respecting participants’ perspectives and preferences for lifestyle change. This delivery style aims to promote intrinsic interest and foster sustained engagement among participants. The coaches learn to provide a rationale for behaviour change, to collaboratively develop behaviour change options for the men to choose from, and to facilitate the development of participants’ personally-relevant goals (rather than imposing goals on them). Engagement is promoted by ensuring the sessions are enjoyable, fun, non-dogmatic, experiential and interactive. Positive banter is encouraged to create a mutually supportive ‘team’ environment that helps men to learn from each other by sharing tips and advice, whilst facilitating interactional styles that men are familiar with in other (predominantly) male contexts [26]. Importantly, the program aims to maximise the time spent interacting with peers to promote long-term behaviour changes through the collaborative construction of changes to masculine identities and the ways they are expressed [15, 23].

Positive feedback and celebration of individual progress (not just achievement) towards small, short-term goals [19] helps participants feel competent and confident that they can succeed in their long-term physical activity, sedentary behaviour, healthy eating targets (as well as weight loss, if appropriate). Drawing on Self-Determination and Achievement Goal theories and the process evaluation of FFIT, men are also encouraged to recognise the personal value and benefits of the changes that they are making (e.g. feeling fitter, having more energy) [18]. Throughout the program, long-term social support [27] is promoted within the group by encouraging positive interactions to build relationships during the 12 weekly sessions and by encouraging the men to use social media (i.e. WhatsApp, Facebook, etc.) and the MatchFIT app to support each other outside the sessions and to meet up between sessions to exercise together, as well as by encouraging the men to enlist the support of their wider social networks (e.g. family, friends). The light-touch reunion session (6–9 months after the start of the program) provides men with an opportunity to share their experiences of maintaining the changes they made during the program since the end of the initial 12, weekly sessions.

### Comparison group

As a waiting list control group, the comparison group will be placed on a wait list to be offered a guaranteed place on the EuroFIT program after their 12 month follow-up measurements are completed. In addition, all men (both intervention and comparison group) will receive a healthy lifestyle leaflet following the baseline measurement and prior to randomisation. These leaflets will be selected on a country by country basis, with the criteria for selection being that they promote forms of physical activity that are widely appropriate in their own country and include country-specific physical activity guidelines.

## Data collection

### Recruitment

Men will be recruited through the following clubs:

- The Netherlands: ADO Den Haag; FC Groningen; PSV; Vitesse.
- Norway: Rosenborg BK; Strømsgodset IF; Vålerenga Fotball.
- Portugal: Futebol Clube do Porto; Sporting Clube de Portugal; Sport Lisboa e Benfica.
- UK (England): Arsenal FC; Everton FC; Manchester City FC; Newcastle United FC; Stoke City FC.

Participants will be recruited from June 2015 onwards in the Netherlands, Portugal and the UK (England). Due to the later start of the football season, recruitment in Norway will be from November 2015 onwards. Each of the 15 clubs across the four countries will recruit up to 100 interested men who will be invited to an initial visit to the club to check eligibility. We aim to include a total of 1000 participants for the trial.

We will use different recruitment strategies matching individual clubs’ preferences. These may include club-based activities, such as online publicity (e.g. advertising on club/fan websites), e-mail, newsletter or social media announcements (i.e. Twitter, Facebook), poster/flyers, end of season home match-day advertising, face-to-face recruitment at home matches (handing out leaflets and collecting contact details), active involvement of local supporters’ organisations and word of mouth. We may also, where appropriate, publicise the program on national football league websites and try to gain media publicity via newspapers (local, regional, national), radio and TV coverage. In addition, national EuroFIT websites will be developed to attract men and provide information about participation in the trial.

Men will be able to register their interest online through a provided link (developed for the study and linked to the study database). The research team will then phone all men who have registered an interest in taking part in EuroFIT as part of the trial (the only way in which the EuroFIT program will be available at this time). The researchers will discuss the study and conduct an initial telephone screening for eligibility by administering the PARQ+ and checking that the man is not already involved in another health-related program being delivered by the club. Eligible participants will be sent a confirmation e-mail or postal letter, including the participant-information sheet, a consent form and an appointment to attend an information meeting at their club. At this information meeting, researchers will explain the study procedures and inclusion criteria, and take men’s written informed consent for taking part in the study. Those who agree to take part in the trial will be asked to indicate in writing whether they are willing to provide optional blood samples. At the club visit, participants will be asked to sign the PAR-Q+ screening instrument that was previously administered over the telephone. Men who have provided informed consent to take part in the trial will be fitted with an activPAL activity monitor to wear for the next seven days. Participants who provide at least four days of valid data (at least 10 h per day of activPAL data) as assessed at a return visit to the club one week later, will be included in the study. They will then complete the remaining baseline assessments and proceed to randomisation. Participants with less than four days of valid activPAL data will be asked to wear the activPAL for another 7 days or will be excluded from participation in the study [28].

Participants can leave the study at any time for any reason and without consequences. Intervention group participants who drop out from the EuroFIT program will still be invited to attend follow-up measurement sessions as part of the trial. Participants who cannot attend or fail to show up for their follow-up measurement appointment at the club will be offered a home measurement visit or visit to the university premises to maximise retention to the trial. If participants wish to fully withdraw from the study, their reason for leaving the study will be obtained via a structured phone interview, where possible.

All participants will be offered club vouchers for attending follow-up measurement appointments (post-program follow-up: 25 euro/20 pounds/400 kroner; 12 month follow-up 75 euro/60 pounds/600 kroner), as a gesture of thanks for their time commitment. All participants will be offered a short feedback report after the 12 month measures which summarises their changes on key outcomes over the course of the trial.

### Randomisation

We will be using an individually randomised design, as was used in the FFIT RCT, which confirmed that the higher sample size and costs associated with a cluster randomised design were unwarranted as minimal contamination was observed between intervention and comparison group participants (the mean difference in weight loss between groups adjusted for baseline weight and club was 4.9 kg [95 % CI 4.0,5.9]; a sensitivity analyses adding club as a random effect adjusted for baseline weight to account for possible clustering gave 4.9 kg [95 % CI 3.8,6.0]) [14, 29].

Participants will be randomly allocated to the EuroFIT intervention group or the waiting list comparison group in a 1:1 ratio, stratified by football club. The method of randomised permuted blocks will be used, with random block lengths (4 or 6). The randomisation schedule for each club will be generated by a computer program and stored within the Clinical Trials Unit, with access restricted to those responsible for maintenance of the randomisation system. Research staff in each country will not have access to randomisation codes during baseline data collection; when baseline data have been collected, local research staff will access the random allocation for each individual via a study web portal. Data management and statistical staff within the Clinical Trials Unit will not have access to randomisation codes prior to database lock, with the exception of statistical staff providing reports to the Independent Data Monitoring Committee; these staff members will not be involved in the development and implementation of the final statistical analyses.

### Blinding

Because men will know which arm of the study they are in, blinding is not possible. However, because randomisation occurs later, group allocation will not be known to either participants or field staff at baseline assessment. The primary outcomes for the trial will be measured by and downloaded directly from the activPAL, which gives an objective measurement of activity pattern that is not accessible to either research staff or participants until it has been processed. The researchers who process activPAL data will be blind to group allocation.

### Procedures

We will collect data at baseline, and at follow-up assessments immediately post-program and 12 months after baseline. At six months, participants will be asked to complete an additional short online questionnaire for the economic evaluation. Full details of the measures are provided below and the timing of each measurement is provided in Table 1.

**Table 1:** Summary of measures used in the EuroFIT trial

	Baseline	Post-program	6 Months	12 Months
Objective physical activity and sedentary time				
activPAL^tm^ micro	X	X		X
activPAL wearing diary (sleep, work time)	X	X		X
Self-reported behaviours				
Food intake (adapted DINE)	X	X		X
Physical activity (IPAQ-short)	X	X		X
Domain specific and total sedentary time (Marshall)	X	X		X
Sleeping time	X	X		X
Standing time	X	X		X
Sedentary/active behaviours (Activity Choice Index)	X	X		X
Smoking	X	X		X
Objective physical measures				
Body height	X			
Body weight	X	X		X
Waist circumference	X	X		X
Resting blood pressure	X	X		X
Blood biomarkers	X			X
Self-reported health and psychosocial measures				
Wellbeing (Cantril ladder)	X	X		X
Self-esteem (Rosenberg)	X	X		X
Vitality	X	X		X
Quality of Life (EQ-5D-5 L)	X	X		X
Long standing illness, disability or infirmity	X	X		X
Joint pain	X	X		X
Injuries	X	X		X
Self-reported socio demographic moderators				
Age	X			
Ethnicity	X			
Marital status	X			
Education	X			
Current employment status	X			
Income	X			
Self-reported mediators				
Motivation for physical activity (adapted BREQ-2)	X	X		X
Ego/Task involvement	X			X
Club identification (Sport Spectator Identification Scale)	X	X		X
Weight management strategies	X	X		X
Weight loss activities	X	X		X
Self-reported mediators (intervention group only)				
Need support of coach		X		
Need thwarting by coach		X		
Mastery/performance climate		X		
Relatedness to group		X		
Need satisfaction from physical activity				X
Self-reported cost-effectiveness				
Health-related quality of life (EQ-5D-5 L)	X	X	X	X
Health care use (iMTA)		X	X	X
Consequences for employment (iPCQ)		X	X	X
Medication use (iMCQ)	X			X
Travel costs to club	X	X		X
Self-reported process evaluation (intervention group only)				
Coaches	X	X		
Participants	X	X		X

Measurement sessions will be held at the football clubs during evenings, in order to maximise attendance of participants. All measurements will be conducted by researchers/fieldworkers trained by study staff to standardised protocols. Men who opt into the blood testing will have a venous blood sample taken using trained nurses/bioengineers. Participants will be asked to complete a questionnaire (either paper-based or on a tablet provided by the research team). Sufficient staffing will be provided at measurement sessions to allow assistance to be available for men with low literacy or other difficulties in completing the questionnaire. In line with best practice, country validated versions of the questionnaire will be used when available. For parts of the questionnaires lacking official validation, translation will be done by members of the EuroFIT research teams and back-translated into English by the principal investigators in each country.

#### Fieldwork staff training

Fieldwork staff training will be standardized and quality assured. We will organise a training meeting for research leads from each country who will then train the fieldworkers locally. Standard operating procedures will describe all aspects of trial delivery including specification of equipment used in the measurement sessions and any adaptations to survey instruments that are necessary in different country/cultural settings.

#### Measurement feasibility study

The baseline and post-program measurement protocols have been tested during a feasibility study that was conducted between September 2014 and February 2015 in all four participating countries (1 club in the Netherlands, Norway and UK, and 2 clubs in Portugal). In total, 57 men participated in the feasibility study. Lessons learned were incorporated into the final study protocol.

#### Procedures to maximise retention to the trial

To maximise retention at the follow-up assessments we will:

- Send men an advance reminder that follow-up measurements are upcoming, using a personalised letter/e-mail sent 2–4 weeks ahead of the measurement dates at their club;
- Phone men two weeks before the scheduled post-program and 12 month measurement sessions to arrange an appointment time for the measurements;
- Send a confirmation of the date, time and location of the man’s appointment by e-mail/mail (according to men’s individual preferences);
- Text men in the days leading up to their appointment to remind them about the time, date and location;
- Offer men who do not show up at first measurement visit a second opportunity for measurement at the club;
- Offer men a home/university visit if they cannot attend or fail to attend the follow-up assessments at the club;
- Offer men who have successfully completed a follow-up assessment, a club voucher in appreciation of their time.

#### Primary outcomes: objective physical activity and sedentary time

The primary outcomes in this trial are changes in total physical activity (i.e. steps per day) and total sedentary time (i.e. minutes per day spent sitting). This will be objectively assessed with the activPAL activity monitor (model activPAL^TM^ micro; PAL Technologies Ltd., Glasgow, UK). The activPAL is a small monitor that weighs 9 g and is taped to the front of the thigh ideally for at least seven complete consecutive days. It has no display screen; hence the data recorded by the activPAL are not visible without being downloaded and processed. The activPAL has been found to have good measurement properties to assess sitting, standing, stepping and postural transitions in adults [30–32].

Once consent is obtained at the information meeting, trained researchers/fieldworkers will provide participants with face-to-face instruction on how to affix the activPAL to the thigh. The face-to-face instruction will be supported by written guidance on how to fit the activPAL. Participants will be asked to wear the device 24 h per day (including while taking a shower) for seven consecutive days; they will be advised that they should only temporarily remove the device during water submersion activities (e.g. having a bath, swimming) and to refit the device as soon as possible afterwards. Participants will be asked to keep a monitoring log to note any times when the device was removed and replaced. Participants will also be asked to record work and sleep times in the monitoring log. At baseline, the activPAL will be returned when the participant attends the baseline measurement session at the club. At both post-program and 12 month follow-up assessments, participants will receive the activPAL and written instructions by mail for fitting and wearing the device ten days before the follow-up measurement is scheduled at their club. Each participant will receive a reminder text message to remind them to wear the device. Mail delivery of the activPAL was successfully trialled in the feasibility study.

In order to meet the inclusion criteria for the trial, as described above, participants need to provide at least four valid days of activPAL data at baseline. Data from the attachment and removal day will not be used for analyses as these are incomplete days where the participant started or finished wearing the activPAL during the day. ActivPAL data will be considered valid when the participant wore the device for at least 10 h of the waking day.

#### Secondary outcomes

##### Self-reported behaviours

Using an adapted version of the Dietary Instrument for Nutrition Education (DINE) questionnaire [33], we will assess self-reported dietary behaviour via the frequency of intake of the following foods and drinks: cheese, burgers or sausages, beef, pork or lamb, fried food, chips or French fries, bacon or ham or pate, savoury pies, pasties, sausage rolls and pork pies, savoury snacks, consumption of fruit, vegetables (not potatoes), chocolate, sweets, biscuits, sugary drinks (fizzy drinks, diluting/ fruit juice) and milk. We will also assess frequency of breakfast consumption and alcohol consumption.

Self-reported physical activity will be recorded using the International Physical Activity Questionnaire (IPAQ), which assesses walking, other moderate intensity physical activity and vigorous intensity physical activity [34]. Self-reported sedentary time will be assessed with the Marshall questionnaire [35], which assesses total and domain specific sitting time (i.e. sitting during transport, at work, while watching TV, while using the computer for leisure, and during other leisure activities). We will assess both sleeping and standing time using a single item question (*How many hours in each 24 h day do you usually spend: Sleeping (including at night and naps);* or *Standing* [36]). We will capture activity and sedentary behaviours by using the Activity Choice Index [37], measured on a 5-point scale (from ‘never’ to ‘always’). Items include: using stairs instead of escalators or lifts; walking instead of driving or taking public transport; parking away from destination or getting off public transport early to have a longer walk; using work breaks to be physically active; choosing to stand up instead of sitting; choosing to do things by hand instead of using mechanical/automatic tools.

In addition, smoking behaviour will be assessed, including date of quitting and amount of current consumption, when relevant.

##### Objective physical measures

Body height will be measured (to the nearest 1 mm) using a portable stadiometer (Leicester Height Measure) at baseline only after participants have removed their shoes. Body weight will be assessed at all measurements (to the nearest 0.1 kg) using a calibrated electronic flat scale (Tanita HD366). Participants will be allowed to wear light clothes (such as shorts and t-shirts), but will be asked to remove any heavy items of clothing, their shoes and any items in their pockets. We will calculate BMI as weight in kilograms divided by the square of height in metres (kg/m^2^). Waist circumference will be measured twice (to the nearest 0.1 cm) with a Seca 201 measure, with participants asked to remove their shirts. If the difference between the two waist measures is more than 0.5 cm, a third measurement will be conducted. The mean will be calculated from the two nearest measures.

Resting blood pressure will be measured with an Omron 705-CPII blood pressure monitor after 5 min sitting still. If measured systolic blood pressure is over 139 mmHg and/or measured diastolic blood pressure is over 89 mmHg, two further measures will be taken and recorded, and in line with duty of care, men will be given letters advising them to consult their GP. A mean will be calculated from the second and third measures.

Blood samples will be taken at baseline and after 12 months from those who provide the additional consent for this measure. Participants who have opted-in to provide blood samples will be asked to confirm that they have fasted for at least 6 h. Time of last food/drink (other than water) intake will be recorded on the electronic Case Report Form (eCRF). A venous blood sample (using 1 × 9 ml Ethylenediamine Tetraacetic Acid (EDTA) tube, 1 × 7 ml Serum-separating tubes (SST), and 2 × 2 ml fluoride oxalate) will be taken by a trained phlebotomist (usually a fieldwork nurse) using a standard operating procedure. Samples will be stored at 4 °C (either in a refrigerator, cool bag with ice pack or on wet ice) until processing at a local hospital, laboratory, or onsite within 24 h (ideally within 12 h) (42). Two 1 ml aliquots of whole blood from the EDTA tube will be dispensed into barcoded screw-cap Eppendorf tubes. All blood tubes will then be centrifuged at 3000 rounds per minute for 20 min at 4 °C to separate red cells /plasma/serum. The SST will be allowed to clot for at least 30 min after collection before spinning. After spinning, 0.5 ml aliquots will be pipetted with plasma (5 from EDTA tube, 2 from fluoride oxalate tubes) and serum (5 from SST). These will be stored in barcoded tubes at −80 °C in barcoded boxes in an alarmed freezer, with capability to transfer samples promptly into a spare freezer in the event of freezer breakdown. Time of sample collection, start of sample processing and freezing will be recorded in the eCRF. At the end of baseline collection for each country (except for the UK where they will be delivered directly following baseline collection at each club), all baseline samples will be shipped to the Institute of Cardiovascular and Medical Sciences at the University of Glasgow in a single consignment by using World Courier (http://www.worldcourier.com), where they will again be stored at −80 °C. Similarly, the 12-month blood samples will be shipped to Glasgow in a single consignment after all these samples have been collected in each country (except for the UK, as described above).

All blood samples will be analysed at the end of the trial. If analysis of the blood data shows a high risk for any of the cardio-metabolic disease biomarkers that the participant should be aware of, we will inform the participant.

##### Self-reported health and psychosocial measures

Participants will be asked to complete measures of their self-reported health and psychosocial measures, using existing and validated measures were available. Wellbeing will be measured using the Cantril ladder [38]. Self-esteem will be assessed by the 10 item version of the Rosenberg self-esteem questionnaire [39], in which participants rate each statement on a 4-point Likert scale (ranging from ‘strongly agree’ to ‘strongly disagree’). Vitality [40] will be measured using four statements (i.e. ‘I felt alive and vital’; ‘I had energy and spirit’; ‘I nearly always felt alert and awake’; and ‘I felt energised’) on a 7-point scale (ranging from ‘not at all true for me’ to ‘very true for me’). Health-related quality of life will be measured using the EQ-5D-5 L [41]. This is a standardised instrument for use as a measure of health outcomes. Participants rate their mobility, self-care, usual activities, pain/discomfort and anxiety/depression on a 5-point scale. They also rate their health today on a scale from 0 to 100.

In a face-to-face structured interview with a member of the fieldwork staff, participants will be asked to report joint pain, and any long standing illnesses, disabilities or infirmities. Injuries that occurred before and during the EuroFIT trial will also be recorded during this interview.

##### Self-reported socio demographic measures

The self-reported questionnaire will assess demographic characteristics (age, ethnicity, education, marital status, current employment status, income) at baseline. These characteristics will be used as potential moderators of any intervention effects on behavioural and other outcomes, to identify whether the program is more or less beneficial for pre-specified subgroups of men.

##### Self-reported mediators

Motivation for physical activity will be assessed using the adapted Behavioural Regulation In Exercise Questionnaire (BREQ-2) [42]. This questionnaire consists of 15 statements which require a response on a 5-point scale (range ‘not true for me’ to ‘very true for me’); these assess participants’ intrinsic motivation, identified regulation, introjected regulation, external regulation and amotivation in relation to exercise. Participants will also complete six items related to ego/task involvement [43–45], allowing us to explore participants’ motivational criteria for what it takes to succeed according to their own goals. These will be used in part to compare the variance between those that engage with the program to those who do not. The self-reported questionnaire also includes the Sport Spectator Identification Scale which contains seven Likert-scale items assessing identification with a sports team (response options range from 1 (low identification) to 8 (high identification)) to measure men’s degree of identification with their football club [46].

To assess the potential contribution of other weight loss activities, we will ask participants to report if they did anything else to lose weight (such as attending exercise workouts, attending a commercial weight loss program, having weight reduction surgery). Participants will also be asked to report what sort of strategies (i.e. eating breakfast on a daily basis, limiting quantity, restricting intake of certain foods, drinking fewer sugary drinks or less alcohol and consciously eating more slowly) they use to manage their weight on a 5-point scale ranging from ‘never’ to ‘always’.

We will also assess the extent to which EuroFIT participants report that coaches and other group members were able to create a needs-supportive motivational climate. Specifically, we will measure the extent to which participants report that coaches were able to support their autonomy, competence to make changes, and feelings of relatedness, using a 5-point scale ranging from ‘not true for me’ to ‘very true for me’ [47]. We will also measure ‘thwarting’ of autonomy, competence, and relatedness needs by the coach using a 9-item measure adapted from Bartholomew et al. (2011) which are rated on a 7-point scale ranging from ‘strongly disagree’to ‘strongly agree’ [48]. In addition, six items will assess the extent to which men feel the group climate supported mastery and performance rated on a 7-point scale ranging from ‘not at all true’ to ‘very true’ [49]. Relatedness need satisfaction *from the group* will be measured by 6-items adapted from Van den Broeck et. al. (2010), and rated on a 7-point scale ranging from ‘not at all true’ to ‘very true’ [50]. Finally, at 12 months only, we will ask participants to rate the satisfaction they experienced from engaging in physical activity on a 6-point scale (range ‘false’ to ‘true’) drawn from the adapted psychological needs satisfaction in exercise scale [51].

### Economic evaluation

This study will include an evaluation of the cost-effectiveness of EuroFIT in comparison to receipt of a healthy lifestyle leaflet only at 12 months (short-term within trial) and a projected 5 years (long-term modelling) with regard to the primary outcomes of the trial and quality of life.

A Health and Personal Social Service perspective, as well as a societal perspective, will be employed. Data on resource utilisation will be collected using self-report questionnaires based on the iMTA Productivity Cost and Medical Consumption questionnaires (iPCQ and iMCQ) [41] and will include utilisation of healthcare services, medication use and absenteeism from paid work. Medication use will be measured at baseline and after 12 months. Utilisation of healthcare services and absenteeism from paid work costs will be measured post-program, at 6 months and at the 12-month follow-up. Researchers will send participants an email to ask them to complete the 6-month questionnaire online. Men who do not have an email address will be sent a paper version of the questionnaire by post and a reply-paid envelope.

### Process evaluation

A process evaluation will be embedded in the RCT to provide insight into whether the EuroFIT program is delivered as intended, why it did or did not produce the intended outcomes, and how such a program can be delivered within the specific context of a professional football club in future. We will investigate the processes that are necessary for implementation of the EuroFIT program, the way in which the program operates on outcomes and on any unintended outcomes, and participants’ and coaches’ experiences of the program. At both post-program and 12 month follow-up measurements, the intervention group will be asked to complete self-report questions focusing on: experiences with the program; SitFIT and MatchFIT usage; which elements of EuroFIT they found most useful; the extent to which they are still interacting with their EuroFIT peers after the program has ended; and the perceived impact of the EuroFIT program on their lives. The design of the process evaluation is described in a separate study protocol.

### Database

The Robertson Centre for Biostatistics (RCB), University of Glasgow, within the Glasgow Clinical Trials Unit, will oversee RCT data management.

All trial subject data will be entered locally in each country via the study web portal, which features an online data entry system with data validation checks built in. Data will be entered either via a web-portal or a tablet app. All data will be stored securely on the local servers of the RCB. All personal data will be anonymised and encrypted; it will only be possible to link back to an individual via a separately-stored encrypted coding system. Paper data and identifiers will be held in secure locked locations on local University premises. At the end of the trial, final data validation checks will be carried out prior to database locking and unblinding of random group assignment.

### Data analyses

The RCB will provide statistical services in support of delivering the RCT. A detailed Statistical Analysis Plan will be developed, and finalised prior to database lock and unblinding. RCB statisticians will develop analysis programs during the trial, whilst blind to randomised allocations, and communicate any data anomalies to RCB data managers and to the project team at each site for checking.

#### Sample size calculations

The study is powered to detect small, but clinically relevant, changes in the primary outcomes: objectively measured (using the activPAL device) total physical activity (e.g. daily step count) and total sedentary time at 12 months.

Both primary outcomes are powered (separately, not as a composite primary outcome) at 90 % to detect an effect size of 0.25 standard deviation (SD) units at a 2.5 % significance level. In relation to physical activity, which has a standard deviation of approximately 4000 steps per day, the trial will be powered to detect an average increase of at least 1000 steps per day (roughly equivalent to 10 min of moderate intensity physical activity on average per day). In relation to sedentary time, which has a SD of almost 100 min/day [52], the trial is powered to detect an average decrease of at least 25 min per day spent sitting. A sample size of 400 per group will allow us to detect these changes. To achieve this sample size at 12 months we estimate that we will need to include a total of 1000 eligible participants in the RCT.

In relation to blood-based biomarkers, we estimate that >70 % of participants will opt-in to blood sampling (>245 in each arm), which will provide 90 % power, at 5 % significance, to detect an effect size of 0.29 SD units for a normally-distributed outcome measure. Fasting insulin is the biomarker of principal interest; its SD in non-diabetic adults is ~6 mU/l (87), thus the study will be powered to detect a change in fasting insulin with the EuroFIT intervention of at least ~1.7 mU/l.

#### Effectiveness analysis

In accordance with the study aims, statistical analyses will be conducted to determine whether the intervention group differs from the comparison group in changes over time in primary and secondary outcomes. The key analysis will be undertaken on an intention-to-treat basis, regardless of individual engagement with the EuroFIT program. However, further sensitivity analyses will determine the association between attendance at intervention sessions and effectiveness. Analysis of outcomes at each time point will use linear mixed effects regression methods (normal, logistic or other generalised linear models, as required), including random effects for country and football club, and fixed effects for study group and baseline measurement of the outcome. Regression models will be extended to assess moderators and mediators of intervention effects. The patterns and extent of missing data will be examined and, if necessary, methods such as multiple imputation will be implemented to provide robust results for primary and main secondary outcomes, assuming data are missing at random.

### Cost-effectiveness analyses

Cost-effectiveness of the EuroFIT program in comparison with the comparison group will be evaluated at 12 months follow-up. Further, using data from the randomised controlled trial and epidemiological data, the long-term cost-effectiveness of the EuroFIT program in comparison with the comparison group will be modelled over a period of 5 years.

#### 12-month cost-effectiveness

Incremental Cost-Effectiveness Ratios (ICERs) will be calculated by dividing differences in costs between the groups by the differences in primary outcomes and Quality-Adjusted Life-Years (QALYs). Differences in costs and effects will be analysed using linear multilevel regression analyses. Clustering at the level of the football club will be included in these multilevel models. To estimate statistical uncertainty, 95 % confidence intervals will be estimated around cost and effect differences using bias-corrected bootstrapping with 5000 replications [53]. To account for the clustering of data, bootstrap replications will be stratified by football club [54].

To graphically illustrate the uncertainty around the ICERs, bootstrapped incremental cost-effect pairs will be plotted on cost-effectiveness planes [55]. A summary measure of the joint uncertainty of costs and effects will be presented using cost-effectiveness acceptability curves (CEACs). CEACs show the probability that the intervention is cost-effective in comparison with the comparison group at different ceiling ratios (i.e. the maximum amount of money decision-makers are willing to pay per unit of effect) [56].

#### 5-year cost-effectiveness

The longer term analysis will employ a health economic model, populated with data from published literature, to link the short term health outcomes measured within the trial to potential longer term impacts on health (e.g. impacts on the development of cardiovascular disease, diabetes, etc.) and the estimated economic consequences.

Based on the literature, we will estimate:

- To which degree the participants will continue to show the behaviour changes achieved during the EuroFIT intervention;
- How the short-term health outcomes measured within the trial (i.e. to 12 months) link to longer term impacts on health (e.g. in terms of impacts on the development of cardiovascular disease, diabetes, etc.);
- What the costs associated with these health impacts are;
- What the quality of life weights, i.e. utility scores, associated with these health impacts are.

In this analysis, different scenarios will be evaluated in which the retention effect and implementation rate of the intervention will be varied. Probabilistic sensitivity analyses will be employed in which probability distributions are estimated for all inputs into the model. Subsequently, Monte Carlo simulation techniques will be used to estimate uncertainty in the cost-effectiveness analyses. This uncertainty will be shown in cost-effectiveness acceptability curves.

## Discussion

This paper describes the protocol of the EuroFIT RCT, a study designed to assess the effectiveness and cost-effectiveness of a lifestyle intervention program offered by top flight European professional football clubs to their male football fans. A major strength of the EuroFIT program is that it builds on the successful FFIT program which was designed to support overweight and obese men in losing weight [14]. The EuroFIT program is the result of considerable developmental work to extend its focus to targeting improvements in physical activity and sedentary behaviour. In addition, new components based on contemporary motivation theory were integrated into the program to maximize the sustainability of the program. The developmental work also includes integration of entirely novel technology, the newly developed SitFIT activity/sitting monitor, which allows for the unique combination of accurate real-time self-monitoring of both physical activity and sedentary/non-sedentary time. It also incorporates a web and mobile app, designed as part of the EuroFIT study, to encourage social support around physical activity both between sessions and post-program.

The evaluation includes a robust randomised trial design with baseline, post-program and 12-month follow-up measures to assess short- and long-term effects. The RCT design is pragmatic [57], because pragmatic trials tend to produce results that have high applicability (external validity) for participants and decision makers [58, 59]. This will be key for possible future widespread implementation of the EuroFIT program. A cost-effectiveness evaluation will be undertaken alongside the trial to determine whether the program represents ‘value-for-money’ as compared with the comparison group. A process evaluation will provide insights into the role of the different program components in the program’s effectiveness, as well as insight into implementation and the participants’, coaches’ and clubs’ experiences of the program. In addition, possible working mechanisms will be explored by investigating theoretically-based mediators of program effects on changes in health behaviours, and whether changes in lifestyle are themselves mediators of changes in clinically-measured risk factors. We will also assess potential moderators of any intervention effects on behavioural and other outcomes from EuroFIT, to identify any subgroups of men for whom the program is particularly beneficial or not.

Finally, by involving professional football clubs as a key setting to attract the at-risk and hard-to-reach group of middle-aged men from all walks of life to engage in healthier lifestyles, the EuroFIT RCT will provide evidence on the effectiveness and cost-effectiveness of the EuroFIT program delivered by football clubs to their male fans, and will offer insight into the factors associated with successful changes to physical activity and sedentary behaviour, and secondary outcomes, such as diet.

## Abbreviations

BMI, body mass index; BREQ, Behavioural Regulation In Exercise Questionnaire; CEACs, cost-effectiveness acceptability curves; DINE, Dietary Instrument for Nutrition Education; eCRF, electronic Case Report Form; EDTA, Ethylenediamine Tetraacetic Acid; EuroFIT, European Fans in Training; FFIT, Football Fans in Training; ICERs, Incremental Cost-Effectiveness Ratios; iMCQ, iMTA Medical Consumption questionnaires; IPAQ, International Physical Activity Questionnaire; iPCQ, iMTA Productivity Cost questionnaires; PAR-Q+, Physical Activity Readiness Questionnaire-Plus; QALYs, Quality-Adjusted Life-Years; RCB, Robertson Centre for Biostatistics; RCT, randomised controlled trial; SD, standard deviation; SST, Serum-separating tubes.
